# Supplementary material for: Observation of discrete-light temporal refraction by moving potentials with broken Galilean invariance
Source: Nat Commun. 2024 Jun 27;15:5444. doi: 10.1038/s41467-024-49747-3 (PMC11211399; doi:10.1038/s41467-024-49747-3)
Supplement: Supplementary file 1 — Supplementary Information [file 41467_2024_49747_MOESM1_ESM.pdf]

Supplementary Materials for

Observation of discrete-light temporal refraction by moving potentials with broken Galilean invariance

Chengzhi Qin[1,#], Han Ye[1,#], Shulin Wang[1,#], Lange Zhao[1], Menglin Liu[1], Yinglan Li[1], Xinyuan Hu[1], Chenyu Liu[1], Bing Wang[1,*], Stefano Longhi[2,3,*], and Peixiang Lu[1,4,*]

[1] *Wuhan National Laboratory for Optoelectronics and School of Physics, Huazhong University of Science and Technology, Wuhan 430074, China.*

[2] *Dipartimento di Fisica, Politecnico di Milano, Piazza Leonardo da Vinci 32, I-20133 Milano, Italy.*

[3] *IFISC (UIB-CSIC), Instituto de Fisica Interdisciplinar y Sistemas Complejos, E-07122 Palma de Mallorca, Spain.*

[4] *Hubei Key Laboratory of Optical Information and Pattern Recognition, Wuhan Institute of Technology, Wuhan 430205, China.*

[#] C. Q., H. Y. and S. W. contributed equally to this work

[*] Corresponding authors:

B.W. (email: wangbing@hust.edu.cn),

S. L. (email: stefano.longhi@polimi.it),

P. L. (email: lupeixiang@hust.edu.cn).

Supplementary Sections:

Section 1: Floquet band matching analysis and simulation results for several moving speeds.

Section 2: Galilean covariance and rigorous scattering analysis.

Section 3: Experimental results for ac-driving moving gauge-potential barriers.

Section 4: Detailed derivations of Dirac equation and ZB trajectory.

Section 5: Application examples with moving boundaries/potentials.

Section 1: Floquet band matching analysis and simulation results for several moving speeds.

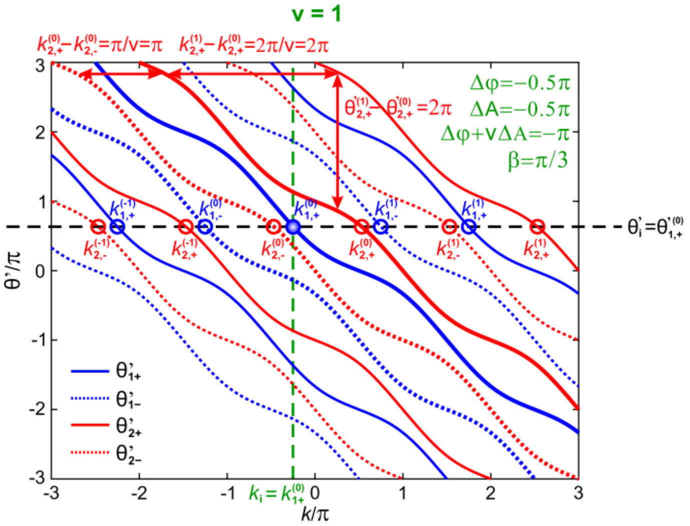

Fig. S1| Floquet bands of *l* = 0, ±1 for permitted integer moving speed *v* = 1. For any incident θ_i', there is only one refracted packet at $k_{2,+}^{(l)}$ or $k_{2,-}^{(l)}$ in each Floquet order. The bolded curves denote the *l* = 0 order. Since $k_{2,\pm}^{(l+1)} - k_{2,\pm}^{(l)} = 2\pi / v = 2\pi$, the packets in adjacent Floquet orders share the same group velocities and show no beam splitting.

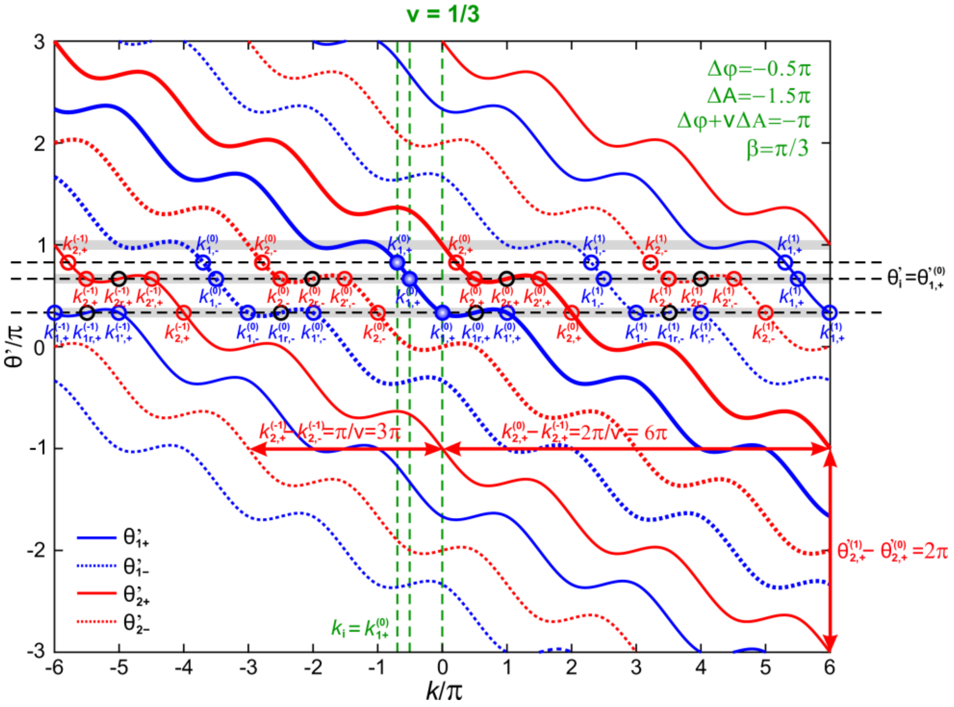

Fig. S2| Floquet bands of *l* = 0, ±1 for permitted fractional moving speed *v* = 1/3. As θ_i' lies in the reflectionless range, there is only one refracted packet at $k_{2,\pm}^{(l)}$ in each Floquet order. As θ_i' lies in the reflective range (gray ribbons), there are two refracted packets at $k_{2,\pm}^{(l)}, k_{2',\pm}^{(l)}$ and one reflected packet $k_{2r,\pm}^{(l)}$

in each order, which generally lead to beam splitting. However, at specific incident θ_i' in the reflective regime, two refracted packets at $k_{2,\pm}^{(l)}, k_{2',\pm}^{(l)}$ happen to share the same group velocities to eliminate beam splitting. For $k_i = -\pi/2$ as an example, we have $v_{g,+}(k_{2,+}^{(0)}) = v_{g,+}(k_{2',+}^{(0)}) = 0$ for $k_{2,+}^{(0)} = \pi/2, k_{2',+}^{(0)} = 3\pi/2$, to show no beam splitting. This beam splitting elimination in the reflective regime can only be fulfilled accidently at one specific θ_i', like the cases in Figs. 3(d) and 3(e).

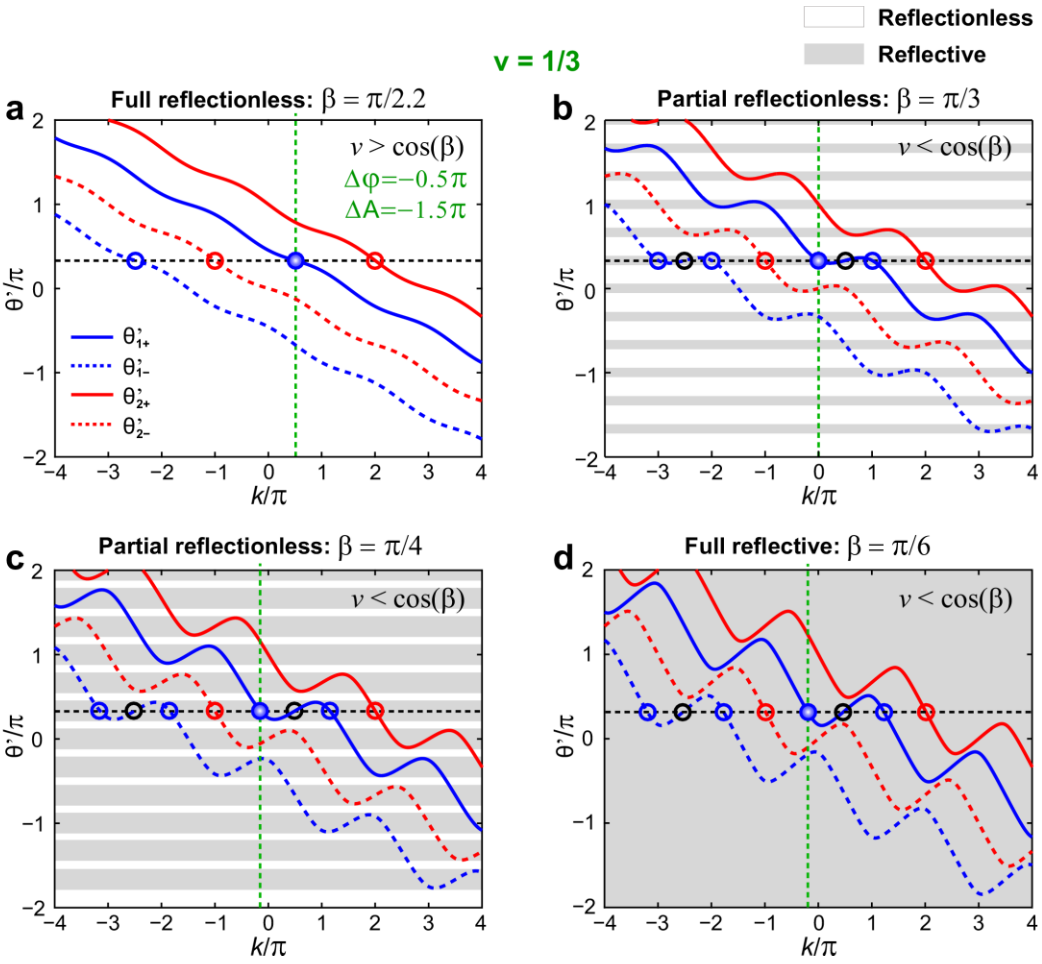

Fig. S3| Size tuning of the reflective range by β for v = 1/3. In the four examples, we choose **(a)** $\beta = \pi/2.2$, (**b**) $\beta = \pi/3$, (**c**) $\beta = \pi/4$ and (**d**)$\beta = \pi/6$. As β decreases, the reflective range is enlarged, from full reflectionless to partial reflectionless, and finally to the full reflective regime. The reason is that cos(β) determines the scope of each Floquet band and hence the size of reflective regime.

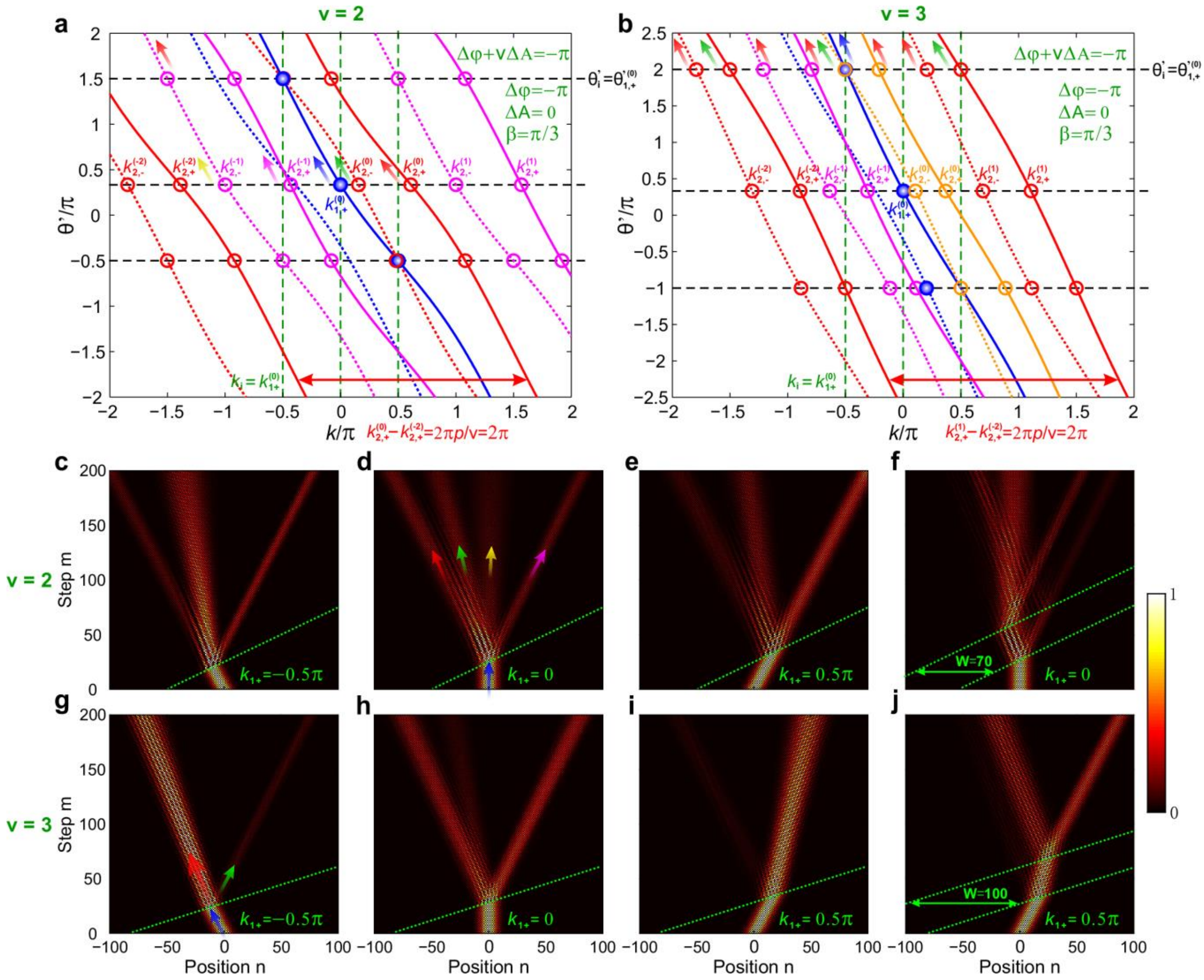

Fig. S4| Floquet bands and simulated beam splitting for unpermitted integer moving speeds v = 2 and v = 3. a, Floquet bands for $v = 2$, with blue curves for $l = 0$ order of incident region, red for l = 0, −2 and magenta for $l = \pm 1$ orders in the barrier. Since $k_{2,\pm}^{(l+2)} - k_{2,\pm}^{(l)} = 2\pi p / v = 2\pi$, there are generally 4 refracted packets at $k_{2,\pm}^{(0)}$ (red) and $k_{2,\pm}^{(-1)}$ (magenta). **b**, Floquet bands for $v = 3$, with red for $l = -2,1$, magenta for $l = -1$ and orange for $l = 0$ order. Since $k_{2,\pm}^{(l+3)} - k_{2,\pm}^{(l)} = 2\pi p / v = 2\pi$, the refracted packets with l spaced by 3 are the same, giving rise to 6 refracted packets at $k_{2,\pm}^{(-2)}$ (red), $k_{2,\pm}^{(-1)}$ (magenta) and $k_{2,\pm}^{(0)}$ (orange). **c-e**, Simulated packet refractions at a moving boundary with $v = 2$ for $k_{1,+} = -0.5\pi$ (**c**), 0 (**d**) and 0.5π (**e**), all showing beam splitting into 3 or 4 branches (denoted by 4 arrows with different colors). **f**, Simulated packet's tunneling through a moving barrier with $v = 2$, also showing beam splitting into multiple beams. **g-j**, Simulated results for $v = 3$, showing beam splitting into 2 (denoted by red and green arrows) or 3 branches, due to very close group velocities among some of them.

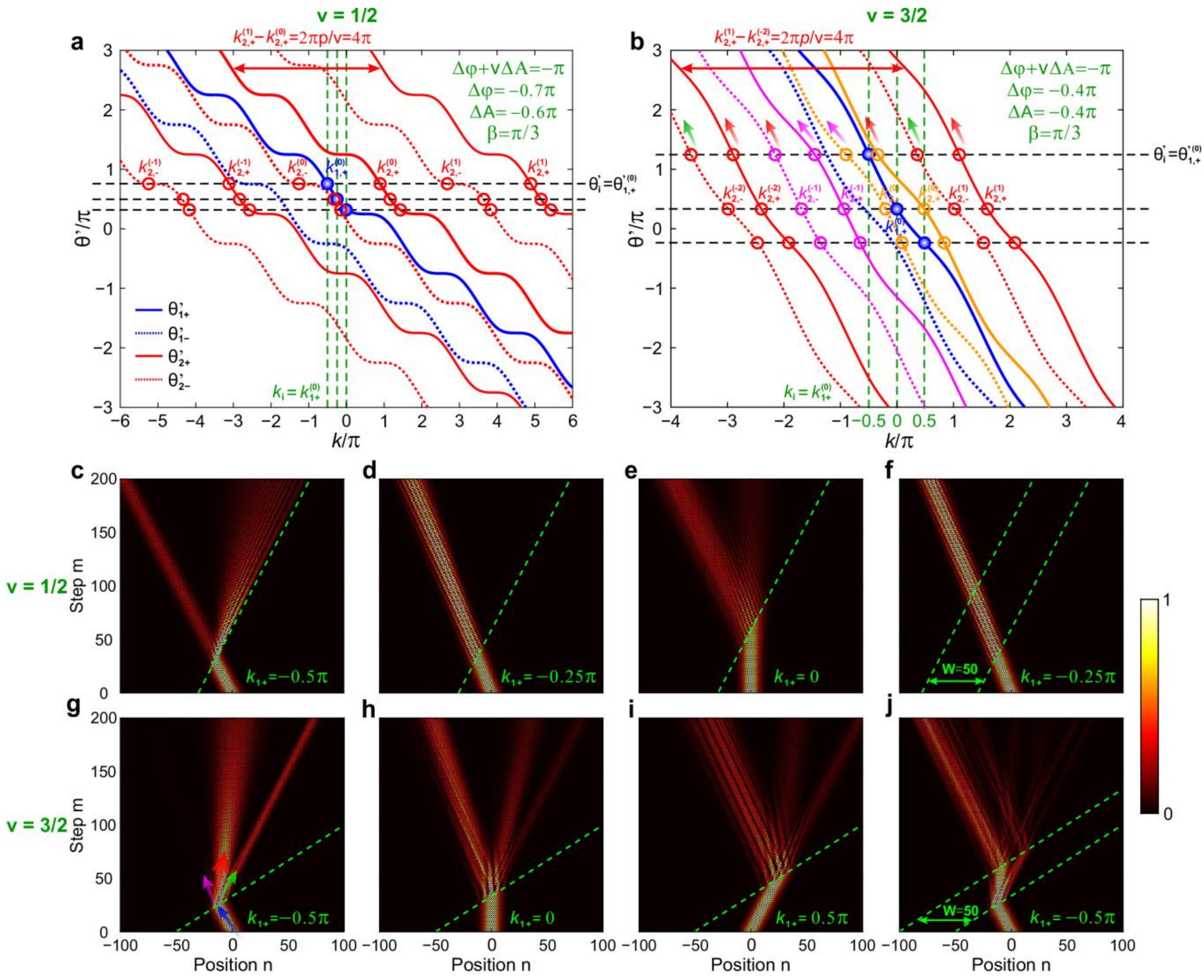

Fig. S5| Floquet bands and simulated beam splitting for unpermitted fractional moving speeds of v = 1/2 and v = 3/2. a, Floquet bands of $l = 0, \pm 1$ for $v = 1/2$, where the blue and red circles denote the incident and refracted packets. Since $k_{2,\pm}^{(l+1)} - k_{2,\pm}^{(l)} = 2\pi p / v = 4\pi$, the adjacent Floquet orders don't contribute to beam splitting, but the refracted packets in "±" minibands within one Floquet order have different group velocities and lead to two splitted beams. **b,** Floquet bands of $l = 0, \pm 1, -2$ for $v = 3/2$. Since $k_{2,\pm}^{(l+3)} - k_{2,\pm}^{(l)} = 2\pi p / v = 4\pi$, both the "±" minibands within each Floquet order and the unequal p Floquet orders contribute to the beam splitting, giving rise to total $2p = 6$ splitted beams. **c**-**e**, Simulated packet refractions for $v = 1/2$ with $k_{1,+} = -0.5\pi$ (**c**), -0.25π (**d**) and 0 (**e**). For $k_{1,+} = -0.5\pi$, there is a grazing refraction beam due to the group velocity $v_{g,-}(k_{2,-}) = 1/2 = \cos(\beta) = v$. **f**, Simulated refraction for $k_{1,+} = -0.25\pi$, showing zero temporal delay $d = 0$. **g-i**, Simulated refraction and tunneling for v = 3/2 under $k_{1,+} = -0.5\pi$ (**f**), 0 (**g**) 0.5π (**h**) and −0.5π (**j**), all showing beam splitting.

Section 2: Galilean covariance and rigorous scattering analysis.

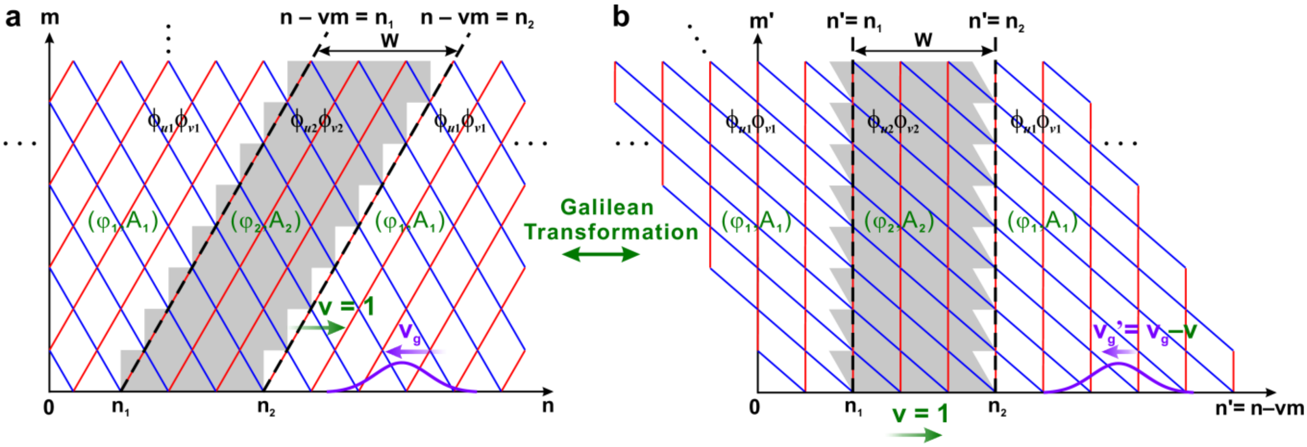

Fig. S6| Schematic of Galilean transformation between (n, m) and (n', m') frames for $v = 1$. a, The lattice model in the laboratory reference frame (n, m). **b,** The lattice model in the moving reference frame (n', m'). The boundaries move one lattice site in one evolution step, corresponds to a moving speed $v = 1$. All other marks are kept the same with $v = 1/3$ case in the main text Fig. 1c.

In this supplementary section, we show that Eqs. (1) are not covariant by a Galilean boost, and provide rigorous analysis of refraction from a moving potential barrier involving both propagative and evanescent waves. By applying Galilean transformation $m' = m$, $n' = n - vm$ in Eqs. (1), we can obtain the coupled-mode equations in the (n', m') frame

$$\begin{cases} u_{n'+vm'}^{m'+1} = [\cos(\beta)u_{n'+vm'+1}^{m'} + i\sin(\beta)v_{n'+vm'+1}^{m'}]e^{i\phi_u(n')} \\ v_{n'+vm'}^{m'+1} = [i\sin(\beta)u_{n'+vm'-1}^{m'} + \cos(\beta)v_{n'+vm'-1}^{m'}]e^{i\phi_v(n')} \end{cases}, \tag{S2-1}$$

where $m' = 0, 1, 2,\dots$ and n' takes a fractional number due to the rational nature of v. As shown in Fig. 1c and Fig. 1d, the coordinates of three coupling nodes (marked by the black circles) are (n, m+1), (n_1, m), (n_2, m) and (n', m'+1), (n'_1, m'), (n'_2, m') in the two frames. Since $n'_2 = n_2 - vm = n+1-vm = n' + v(m'+1)+1-vm' = n'+1+v$, and similarly, $n'_1 = n'-1+v$, we can denote

$$\begin{cases} f(n', m'+1) = u_{n'+vm'}^{m'+1} \\ g(n', m'+1) = v_{n'+vm'}^{m'+1} \end{cases} \tag{S2-2}$$

which leads to

$$\begin{cases} u_{n'+vm'\pm1}^{m'} = f(n'\pm1+v, m) \\ v_{n'+vm'\pm1}^{m'} = g(n'\pm1+v, m) \end{cases} \tag{S2-3}$$

so that the coupled-mode equation of Eq. (S2-1) can be rewritten as

$$\begin{cases} f(n',m'+1)=[\cos(\beta)f(n'+1+v,m')+i\sin(\beta)g(n'+1+v,m')]e^{i\phi_u(n')} \\ g(n',m'+1)=[i\sin(\beta)f(n'-1+v,m')+\cos(\beta)g(n'-1+v,m')]e^{i\phi_v(n')} \end{cases}, \tag{S2-4}$$

Which is given by Eq. (3) in the main text. Clearly, Eqs. (S2-4) depends in a nontrivial way on the drift velocity v, and such a dependence cannot be eliminated by any gauge transformation of functions f and g. This means that Eqs. (1) are not covariant under a Galilean boost, and thus the refraction features of wave packets from a moving potential barrier largely differ from those of the same potential barrier at rest. Breakdown of Galilean invariance can be also inferred by looking at the band structures: only when the band structure is a quadratic function of quasi-momentum k the underlying wave equation displays Galilean invariance. This limiting case can be obtained by assuming a strong coupling angle $\beta \to \pi/2$ and in the continuous (long-wavelength) limit $k \to 0$, for which two independent continuous Schrodinger equations are obtained corresponding to the parabolic approximation of the exact quasi-energy dispersion curves near $k = 0$. However, it should be mentioned violation of Galilean invariance is obtained in our model even in the continuous (long-wavelength) limit $k \to 0$ when we assume a weak coupling $\beta \to 0$. In fact, as shown in the main text in this regime wave dynamics is described by a Dirac-like wave equation, which is covariant for Lorentz (rather than Galilean) boosts.

In the following, we present the exact procedure of solving the scattering problem in the moving reference frame using Eqs. (S2-4). To this aim, let us first consider the uniform lattice case, so that the eigen mode of Eq. (S2-4) is the Floquet-Bloch mode

$$\begin{pmatrix} f(n',m') \\ g(n',m') \end{pmatrix} = \begin{pmatrix} F(n') \\ G(n') \end{pmatrix} e^{-i\theta' m'} = \begin{pmatrix} U' \\ V' \end{pmatrix} e^{ikn'} e^{-i\theta' m'}, \tag{S2-5}$$

where $(U', V')^{\mathrm{T}} = (U, V)^{\mathrm{T}}$ is the eigenstate. By substituting Eq. (S2-5) into Eq. (S2-4), we can obtain

$$\begin{cases} F(n')e^{-i\theta'} = [\cos(\beta)F(n'+1+v)+i\sin(\beta)G(n'+1+v)]e^{i\phi_u(n')} \\ G(n')e^{-i\theta'} = [i\sin(\beta)F(n'-1+v)+\cos(\beta)G(n'-1+v)]e^{i\phi_v(n')} \end{cases}, \tag{S2-6}$$

Let us take the first refraction at right boundary $n' = n_2$ as an example. Rigorously speaking, both propagative wave with real Bloch momenta and evanescent waves with imaginary Bloch momenta can exist in refraction due to completeness requirement of wave-function continuity condition. However, evanescent waves decay into zero by propagating away from the interface and don't contribute to the transmission through the barrier. By involving both propagative and evanescent waves, we can write down the incident, reflected and refracted packets as

$$\begin{pmatrix} F(n') \\ G(n') \end{pmatrix} = \begin{pmatrix} F(n') \\ G(n') \end{pmatrix}_i (n' > n_2) + \begin{pmatrix} F(n') \\ G(n') \end{pmatrix}_r (n' > n_2) + \begin{pmatrix} F(n') \\ G(n') \end{pmatrix}_t (n' < n_2). \qquad \text{(S2-7)}$$

where i, r, t denotes the incident, reflected and refracted waves, which read

$$\begin{pmatrix} F(n') \\ G(n') \end{pmatrix}_i = \begin{pmatrix} U_+(k_{1,+}^{(0)}) \\ V_+(k_{1,+}^{(0)}) \end{pmatrix}_1 e^{ik_{1,+}^{(0)}n'}, \qquad \text{(S2-8)}$$

$$\begin{pmatrix} F(n') \\ G(n') \end{pmatrix}_r = \sum_{l,\alpha,\pm} r_{1p,\pm}^{(l,\alpha)} \begin{pmatrix} U_+(k_{1p,\pm}^{(l,\alpha)}) \\ V_+(k_{1p,\pm}^{(l,\alpha)}) \end{pmatrix}_1 e^{ik_{1p,\pm}^{(l,\alpha)}n'} + \sum_{l,\gamma,\pm} r_{1e,\pm}^{(l,\gamma)} \begin{pmatrix} U_+(k_{1e,\pm}^{(l,\gamma)}) \\ V_+(k_{1e,\pm}^{(l,\gamma)}) \end{pmatrix}_1 e^{ik_{1e,\pm}^{(l,\gamma)}n'}, \qquad \text{(S2-9)}$$

$$\begin{pmatrix} F(n') \\ G(n') \end{pmatrix}_t = \sum_{l,\eta,\pm} t_{2p,\pm}^{(l,\eta)} \begin{pmatrix} U_+(k_{2p,\pm}^{(l,\eta)}) \\ V_+(k_{2p,\pm}^{(l,\eta)}) \end{pmatrix}_2 e^{ik_{2p,\pm}^{(l,\eta)}n'} + \sum_{l,\delta,\pm} t_{2e,\pm}^{(l,\delta)} \begin{pmatrix} U_+(k_{2e,\pm}^{(l,\delta)}) \\ V_+(k_{2e,\pm}^{(l,\delta)}) \end{pmatrix}_2 e^{ik_{2e,\pm}^{(l,\delta)}n'}, \qquad \text{(S2-10)}$$

where $r_{1p,\pm}^{(l,\alpha)}(r_{1e,\pm}^{(l,\gamma)}), t_{2p,\pm}^{(l,\eta)}(t_{2e,\pm}^{(l,\delta)})$ are reflected and refracted coefficient of propagative (with subscript p) and evanescent waves (with subscript e) at $k_{1p,\pm}^{(l,\alpha)}(k_{1e,\pm}^{(l,\gamma)}), k_{2p,\pm}^{(l,\eta)}(k_{2e,\pm}^{(l,\delta)})$, α, γ, η, δ = 1, 2… is serial number.

The explicit evanescent waves are obtained by searching the roots using Eq. (5) of main text in the complex-k plane, i.e., $\cos(\theta_i' + \Delta\varphi + vk_2) = \cos(\beta)\cos(k_2 - \Delta A)$, by letting l = 0. To determine all roots, we create the function from Eq. (5)

$$S(k_2) = \log\left\{\left|\frac{1}{\cos(\theta_i' + \Delta\varphi + vk_2) - \cos(\beta)\cos(k_2 - \Delta A)}\right|\right\}. \qquad \text{(S2-11)}$$

where a propagative wave corresponds to a pole of $S(k_2)$ on the real k_2 axis, while the evanescent wave corresponds to a pole of $S(k_2)$ off the real k_2 axis.

Finally, we outline the general procedure to calculate the coefficients by imposing wave-function continuity relations at some discrete points of n'. If the boundary locates between n' and $n'+1+v$, i.e., $n' \le n_2 \le n'+1+v$, or equally $n_2-1-v \le n' \le n_2$, by applying Eq. (S2-6), we have

$$F(n')_t e^{-i\theta'} = \{\cos(\beta)[F_i(n'+1+v) + F_r(n'+1+v)] + i\sin(\beta)[G_i(n'+1+v) + G_r(n'+1+v)]\} e^{i\phi_u(n')},$$

(S2-12)

Likewise, if the boundary locates between $n'-1+v \le n_2 \le n'$, or equally $n_2 \le n' \le n_2+1-v$, we get

$$[G(n')_i + G(n')_r]e^{-i\theta'} = [i\sin(\beta)F_t(n'-1+v) + \cos(\beta)G_t(n'-1+v)]e^{i\phi_v(n')}, \qquad \text{(S2-13)}$$

For example, for v = 1, Eq. (S2-12) should be satisfied at three points $n' = n_2 - 2$, $n_2 - 1$, n_2, and Eq. (S2-13) is satisfied for $n' = n_2$, which give rise to totally 4 equations. While for v = 1/3, we have $n_2-4/3 \le n' \le n_2$ in Eq. (S2-12), which should be satisfied at $n' = n_2-4/3$, n_2-1, $n_2-2/3$, $n_2-1/3$, n_2; Meanwhile,

we have $n_2 \le n' \le n_2+2/3$ in Eq. (S2-13), which should be satisfied at $n' = n_2$, $n_2+1/3$, $n_2+2/3$. These two cases of $v = 1$ and $v = 1/3$ have been schematically shown in Fig. S6 and Fig. 1c.

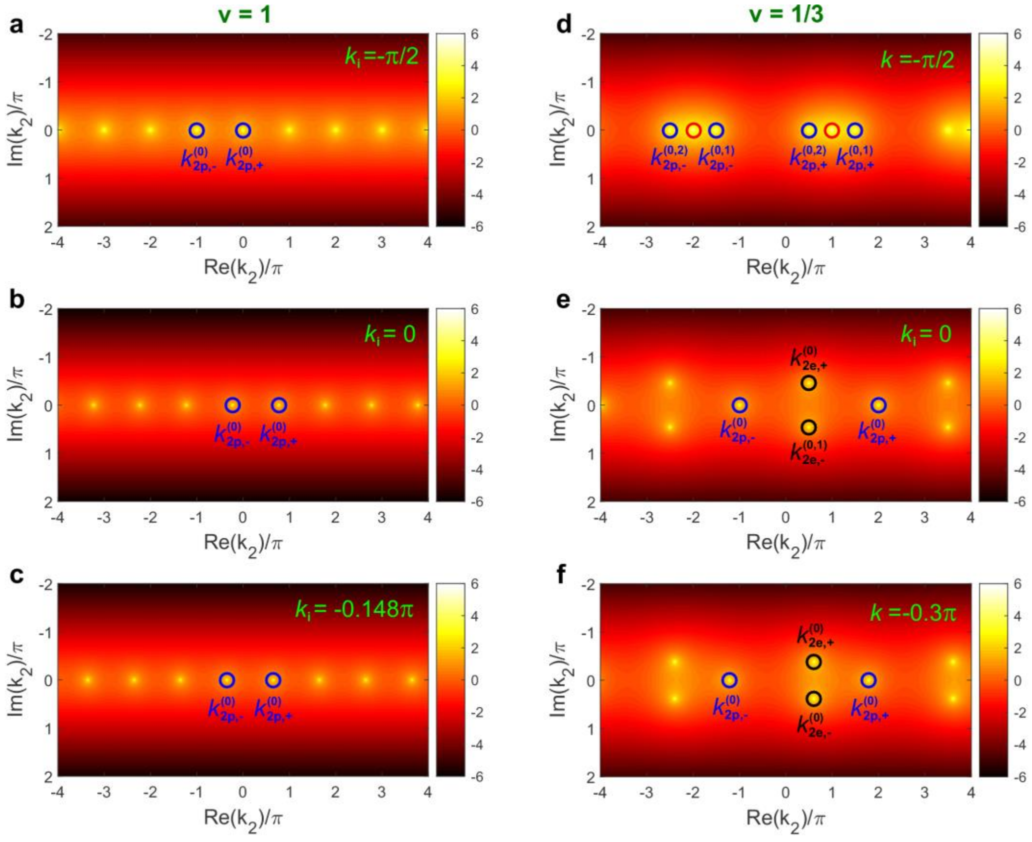

Fig. S7| Numerically-computed propagative and evanescent solutions in the complex-*k* plane. a, b, c. Plotting of the poles $S(k_2)$ of Eq. (S2-11) with $v = 1$, $\Delta\varphi = -\pi/2$, $\Delta A = -\pi/2$, $\beta = \pi/3$ for $k_i = -0.5\pi$, 0 and -0.148π, corresponding to Figs. 3d, 3e and 3g in the main text. Since the poles (denoted by bright spots) all locates on real k_2 axis, the evanescent waves don't exist for $v = 1$. **d, e, f.** Plotting of $S(k_2)$ with $v = 1/3$, $\Delta\varphi = -\pi/2$, $\Delta A = -3\pi/2$, $\beta = \pi/3$ for $k_i = -0.5\pi$, 0 and -0.148π, corresponding to Figs. 4d-4f. For $k_i = -0.5\pi$, there are two propagative waves (blue circles) at $k_{2p,+}^{(0,1)} = 0.5\pi, k_{2p,+}^{(0,2)} = 1.5\pi$ and $k_{2p,-}^{(0,1)} = -2.5\pi, k_{2p,-}^{(0,2)} = -1.5\pi$ in "±" minibands of $l = 0$. Meanwhile, evanescent waves don't exist. For $k_i = 0$, two propagative (blue circles) locate at $k_{2p,+}^{(0)} = 2\pi, k_{2p,-}^{(0)} = -\pi$ and two evanescent waves (black circles) at $k_{2e,\pm}^{(0)} = 0.5\pi \pm 0.4567i$. Likewise, evanescent waves can also occur for the transparent incident condition with $k_i = -0.3\pi$ shown in Fig. S7f.

Section 3: Experimental results for ac-driving moving gauge potential barriers.

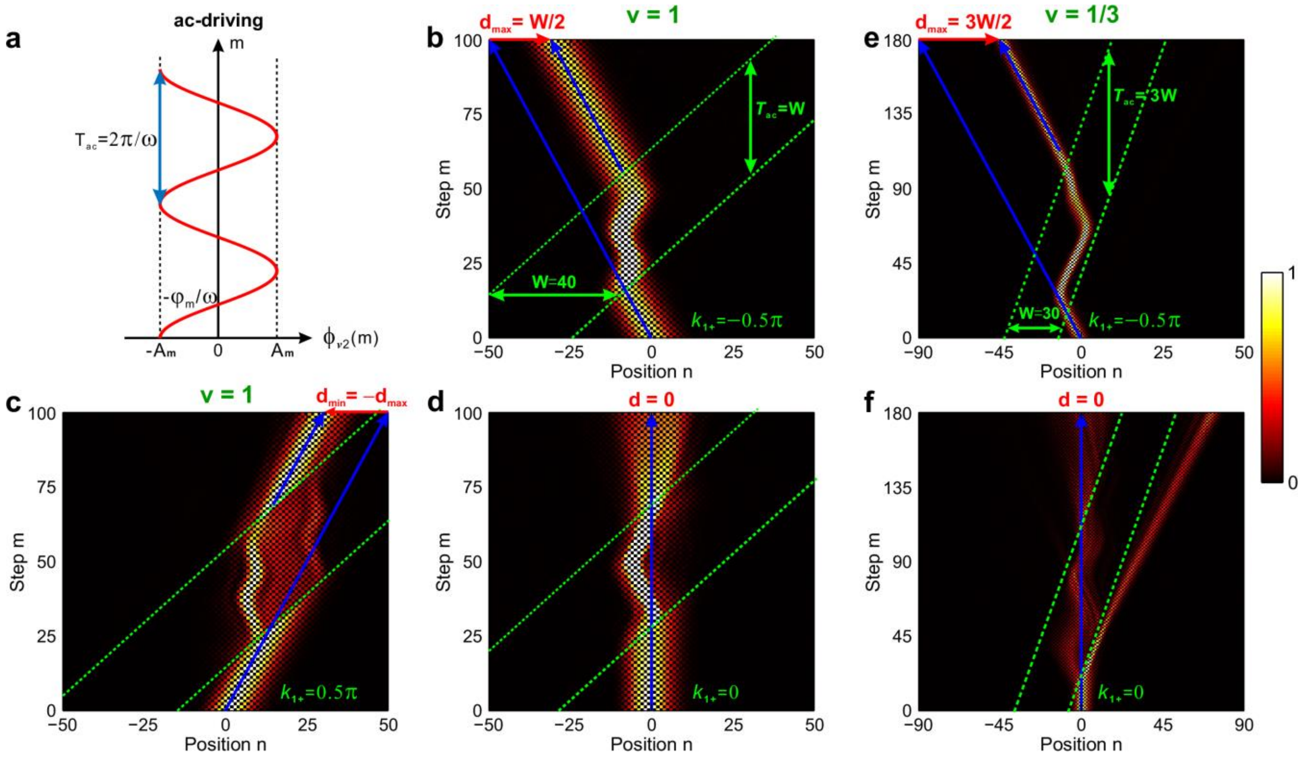

Fig. S8| Measured beam refraction by ac-driving, moving gauge potential barrier. **a**, Schematic waveform of ac-driving phase modulation. **b-d,** Measured field evolutions for $k_{1+} = -0.5\pi$, 0.5π and 0, corresponding to a leftward $d_{\max} = -W/2$ (**b**), rightward $d_{\min} = 2W/2$ (**c**) and $d = 0$ (**d**) for $v = 1$, $A_m = 2.405$, $T_{ac} = W = 40$ and $\varphi_m = 0$. **e**, **f**. Measured field evolutions for $k_{1+} = -0.5\pi$, 0, corresponding to leftward $d_{\max} = -W/2$ (**e**), and $d = 0$ (**f**) for $v = 1/3$, $A_m = 2.405$, $T_{ac} = W/v = 120$ and $\varphi_m = 0.9\pi$.

Section 4: Detailed derivations of Dirac-type equation and ZB trajectory.

In this section, we derive the Dirac-type equation in the weak coupling regime $\beta \to 0$ and calculate the ZB's center-of-mass (COM) trajectory. For a wave packet input at $k = 0$ with slowly-varying envelopes $U(n, m)$ and $V(n, m)$, by denoting $u_n^m = U(n, m)e^{i0n} = U(n, m)$, $v_n^m = V(n, m)e^{i0n} = V(n, m)$, we can rewrite the coupled-mode equations as

$$\begin{cases} U(n,m+1) = \cos(\beta)U(n+1,m) + i\sin(\beta)V(n+1,m) \\ V(n,m+1) = i\sin(\beta)U(n-1,m) + \cos(\beta)V(n-1,m) \end{cases}, \tag{S4-1}$$

Since U, V are slowly varying, both n, m coordinates can be regarded as continuous variables, which lead to $U(n, m+1) = U(n, m) + \partial U(n, m)/\partial m$, $V(n, m+1) = V(n, m) + \partial V(n, m)/\partial m$, $U(n\pm1, m) = U(n, m)$

$\pm$ $\partial U(n, m)/\partial n$ and $V(n\pm1, m) = V(n, m) \pm \partial V(n, m)/\partial n$. By substituting into Eq. (S4-1) and choosing $\cos(\beta) = 1$, $\sin(\beta) = \beta$ in the weak-coupling limit $\beta \to 0$, we can obtain the Dirac-type equation

$$i\frac{\partial \psi}{\partial m} = i\sigma_z\frac{\partial \psi}{\partial n} - \beta\sigma_x\psi, \tag{S4-2}$$

where σ_x, σ_z are Pauli matrices, $\psi(n, m) = [U(n, m), V(n, m)]^T$ is spinor wave function. The ZB's COM trajectory is thus obtained by solving Eq. (S4-2) in momentum space. By performing Fourier transform, we can get

$$\psi(n,m) = \begin{pmatrix} U(n,m) \\ V(n,m) \end{pmatrix} = \int_{-\pi}^{\pi} dk\hat{\psi}(k,m)e^{ikn} = \int_{-\pi}^{\pi} dk\begin{pmatrix} \hat{U}(k,m) \\ \hat{V}(k,m) \end{pmatrix}e^{ikn}, \tag{S4-3}$$

By plugging Eq. (4-3) into Eq. (S4-2), we can obtain the Schrödinger equation describing the evolution of amplitude spectrum $\hat{\psi}(k,m)$

$$i\frac{\partial \hat{\psi}(k,m)}{\partial m} = (-k\sigma_z - \beta\sigma_x)\hat{\psi}(k,m) = H\hat{\psi}(k,m), \tag{S4-4}$$

which can be solved in terms of time evolution operator

$$\hat{\psi}(k,m) = T(m)\hat{\psi}(k,0), \tag{S4-5}$$

where the initial amplitude spectrum is given by

$$\hat{\psi}(k,0) = \frac{1}{2\pi}\sum_{n=-\infty}^{\infty}\psi(n,0)e^{-ikn} = \frac{1}{2\pi}\int_{-\infty}^{\infty}\psi(n,0)e^{-ikn}dn, \tag{S4-6}$$

and the m-dependent transfer matrix is

$$\begin{aligned} T(m) &= e^{-iHm} = e^{i(k\sigma_z+\beta\sigma_x)m} = e^{im\sqrt{k^2+\beta^2}\left(\frac{k}{\sqrt{k^2+\beta^2}},\frac{\beta}{\sqrt{k^2+\beta^2}}\right)\cdot(\sigma_z,\sigma_x)} \\ &= \cos\left(m\sqrt{k^2+\beta^2}\right)I + i\left(\frac{k\sigma_z+\beta\sigma_x}{\sqrt{k^2+\beta^2}}\right)\sin(m\sqrt{k^2+\beta^2}) \end{aligned} \tag{S4-7}$$

where we have utilized the identity $e^{ia(\vec{n}\cdot\vec{\sigma})} = I\cos(a) + i(\vec{n}\cdot\vec{\sigma})\sin(a)$, for an arbitrary variable a and unit vector $|\vec{n}|=1$, By denoting $\sqrt{k^2+\beta^2} = \varepsilon$, Eq. (S4-5) can be rewritten as

$$\begin{aligned} \hat{\psi}(k,m) &= [\cos(\varepsilon m)I + i(k\sigma_z+\beta\sigma_x)\sin(\varepsilon m)/\varepsilon]\hat{\psi}(k,0) \\ &= \begin{pmatrix} \cos(\varepsilon m) + ik\sin(\varepsilon m)/\varepsilon & i\beta\sin(\varepsilon m)/\varepsilon \\ i\beta\sin(\varepsilon m)/\varepsilon & \cos(\varepsilon m) - ik\sin(\varepsilon m)/\varepsilon \end{pmatrix}\begin{pmatrix} \hat{U}(k,0) \\ \hat{V}(k,0) \end{pmatrix} \end{aligned} \tag{S4-8}$$

In our experiment, we input a Gaussian-shaped Bloch-wave packet from the short loop with

$$\psi(n,0)=\begin{pmatrix}U(n,0)\\V(n,0)\end{pmatrix}=\begin{pmatrix}U_0\\V_0\end{pmatrix}e^{-n^2/w_0{}^2}=\begin{pmatrix}1\\0\end{pmatrix}e^{-n^2/w_0{}^2}, \tag{S4-9}$$

where w_0 is the width. Since $\hat{V}(k,0)=0$, we further achieve

$$\begin{cases}\hat{U}(k,m)=[\cos(\varepsilon m)+ik\sin(\varepsilon m)/\varepsilon]\hat{U}(k,0)\\ \hat{V}(k,m)=[i\beta\sin(\varepsilon m)/\varepsilon]\hat{U}(k,0)\end{cases} \tag{S4-10}$$

The packet's COM trajectory of ZB is defined by the expectation value of position operator, which can be calculated in momentum space

$$\begin{aligned}\langle n_{\mathrm{COM}}(m)\rangle&=\int_{-\infty}^{\infty}\psi^*(n,m)n\psi(n,m)dn\\&=2\pi i\int_{-\pi}^{\pi}\hat{\psi}^*(k,m)\partial_k\psi(k,m)dk\\&=2\pi i\int_{-\pi}^{\pi}[\hat{U}^*(k,m)\partial_k\hat{U}(k,m)+\hat{V}^*(k,m)\partial_k\hat{V}(k,m)]dk\end{aligned} \tag{S4-11}$$

The first term of Eq. (S4-11) is calculated as

$$\begin{aligned}&\hat{U}^*(k,m)\partial_k\hat{U}(k,m)\\&=[\cos(\varepsilon m)-ik\sin(\varepsilon m)/\varepsilon]\{\partial_k[\cos(\varepsilon m)+ik\sin(\varepsilon m)/\varepsilon]|\hat{U}(k,0)|^2\\&+[\cos^2(\varepsilon m)+k^2\sin^2(\varepsilon m)/\varepsilon^2]\hat{U}^*(k,0)\partial_k\hat{U}(k,0)\}\end{aligned} \tag{S4-12}$$

in which

$$\begin{aligned}&\partial_k[\cos(\varepsilon m)+ik\sin(\varepsilon m)/\varepsilon]\\&=-\sin(\varepsilon m)m\frac{k}{\varepsilon}+\frac{i\sin(\varepsilon m)}{\varepsilon}+ik[\frac{\cos(\varepsilon m)m\frac{k}{\varepsilon}\varepsilon-\sin(\varepsilon m)\frac{k}{\varepsilon}}{\varepsilon^2}]\\&=-\frac{mk\sin(\varepsilon m)}{\varepsilon}+\frac{imk^2\cos(\varepsilon m)}{\varepsilon^2}+\frac{i\beta^2\sin(\varepsilon m)}{\varepsilon^3}\end{aligned} \tag{S4-13}$$

The second term of Eq. (S4-11) is calculated as

$$\begin{aligned}&\hat{V}^*(k,m)\partial_k\hat{V}(k,m)\\&=[-i\beta\sin(\varepsilon m)/\varepsilon]\hat{U}^*(k,0)\partial_k\{[i\beta\sin(\varepsilon m)/\varepsilon]\hat{U}(k,0)\}\\&=[\beta^2\sin^2(\varepsilon m)/\varepsilon^2]\hat{U}^*(k,0)\partial_k\hat{U}(k,0)\end{aligned} \tag{S4-14}$$

By combing Eqs. (S4-12)-(S4-14), we can obtain

$$\begin{aligned}&\hat{U}^*(k,m)\partial_k\hat{U}(k,m)+\hat{V}^*(k,m)\partial_k\hat{V}(k,m)\\&=[\frac{imk^2}{\varepsilon^2}+\frac{i\beta^2\sin(2\varepsilon m)}{2\varepsilon^3}+\frac{k\beta^2\sin(\varepsilon m)[\sin(\varepsilon m)-\varepsilon m\cos(\varepsilon m)]}{\varepsilon^4}]|\hat{U}(k,0)|^2\\&+\hat{U}^*(k,0)\partial_k\hat{U}(k,0)\end{aligned} \tag{S4-15}$$

which leads to

$$\begin{aligned}\langle n_{\mathrm{COM}}(m)\rangle &= 2\pi i\int_{-\pi}^{\pi}[\hat{U}^*(k,0)\partial_k\hat{U}(k,0)]dk \\ &+2\pi i\int_{-\pi}^{\pi}[\frac{imk^2}{\varepsilon^2}+\frac{i\beta^2\sin(2\varepsilon m)}{2\varepsilon^3}]|\hat{U}(k,0)|^2\,dk \\ &+2\pi i\beta^2\int_{-\pi}^{\pi}\{\frac{k\sin(\varepsilon m)[\sin(\varepsilon m)-\varepsilon m\cos(\varepsilon m)]}{\varepsilon^4}|\hat{U}(k,0)|^2\}dk \\ &=\langle n_{\mathrm{COM}}(0)\rangle-2\pi m\int_{-\pi}^{\pi}(k/\varepsilon)^2\,|\hat{U}(k,0)|^2\,dk \\ &-\pi\beta^2\int_{-\pi}^{\pi}\frac{\sin(2\varepsilon m)}{\varepsilon^3}|\hat{U}(k,0)|^2\,dk+\langle n_{\mathrm{COM}}(m)\rangle_i\end{aligned} \tag{S4-16}$$

The last term of $\langle n_{\mathrm{COM}}(m)\rangle_i$ in Eq. (S4-16) corresponds to an imaginary displacement, which doesn't contribute to a real displacement for ZB and should be neglected. For an incident broad Bloch-wave packet, its spectrum width is narrow around $k = 0$, which can be denoted by $k\in[-\delta k, \delta k]$ with $\delta k \to 0$. In this case, by letting $\varepsilon=\sqrt{k^2+\beta^2}\sim\beta$, we can approximate Eq. (S4-16) as

$$\begin{aligned}\langle n_{\mathrm{COM}}(m)\rangle &= \langle n_{\mathrm{COM}}(0)\rangle-m\frac{2\pi}{\beta^2}\int_{-\delta k}^{\delta k}k^2\,|\hat{U}(k,0)|^2\,dk \\ &-\sin(2\beta m)\frac{\pi}{\beta}\int_{-\delta k}^{\delta k}|\hat{U}(k,0)|^2\,dk \\ &=\langle n_{\mathrm{COM}}(0)\rangle+v_0 m+A_{\mathrm{ZB}}\sin(\omega_{\mathrm{ZB}}m+\varphi_{\mathrm{ZB}}),\end{aligned} \tag{S4-17}$$

where

$$\begin{cases} v_0=-\dfrac{2\pi}{\beta^2}\displaystyle\int_{-\delta k}^{\delta k}k^2\,|\hat{U}(k,0)|^2\,dk=-\dfrac{(\Delta k)^2}{\beta^2}\simeq-\dfrac{1}{(\beta w_0)^2} \\ A_{\mathrm{ZB}}=\dfrac{\pi}{\beta}\displaystyle\int_{-\delta k}^{\delta k}|\hat{U}(k,0)|^2\,dk=\dfrac{1}{2\beta} \\ \omega_{\mathrm{ZB}}=2\beta=\Delta_g,\ (T_{\mathrm{ZB}}=2\pi/\omega_{\mathrm{ZB}}=\pi/\beta) \\ \varphi_{\mathrm{ZB}}=\pi \end{cases}. \tag{S4-18}$$

In above derivation, we have applied the normalization condition of amplitude spectrum

$$\int_{-\delta k}^{\delta k}|\hat{U}(k,0)|^2\,dk=\frac{1}{2\pi}, \tag{S4-19}$$

and the definition of spectrum width in momentum space

$$(\Delta k)^2=\frac{\int_{-\delta k}^{\delta k}k^2\,|\hat{U}(k,0)|^2\,dk}{\int_{-\delta k}^{\delta k}|\hat{U}(k,0)|^2\,dk}\simeq\frac{1}{(w_0)^2}. \tag{S4-20}$$

It shows in Eq. (S4-18) that the oscillation frequency ω_{ZB} is proportional to the coupling angle β while the oscillation amplitude A_{ZB} is inversely proportional to β. Meanwhile, the mean drift velocity v_0 is

inversely proportional to $(\beta)^2$. For a fixed β, v_0 will decrease with the increase of packet's width w_0.

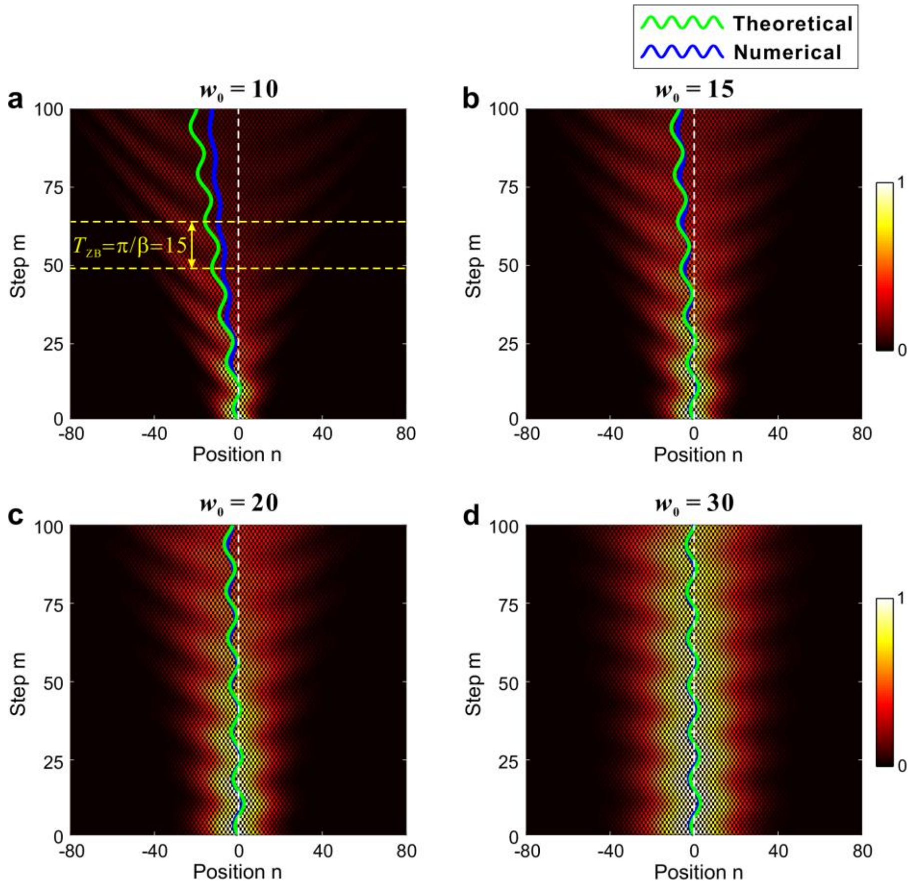

Fig. S9 | Theoretically-calculated and numerically-extracted packet's COM trajectories of ZB. a, **b**, **c** and **d** Simulated field evolutions for the incident packet displaying ZB, showing the characteristic interference patterns of trembling motion for ZB. The green and blue curves denote the theoretically-calculated and numerically-extracted COM trajectories of ZB for several cases of input packet width w_0 = 10, 15, 20 and 30 in **a**, **b**, **c** and **d**. The explicit incident condition is given by Eq. (S4-9).

On the other hand, the COM trajectory of ZB can also be numerically extracted from the simulated amplitude evolutions, which reads

$$\langle n_{\mathrm{COM}}(m)\rangle_{\mathrm{num}} = \frac{\sum_n n\left(|u_n^m|^2 + |v_n^m|^2\right)}{\sum_n\left(|u_n^m|^2 + |v_n^m|^2\right)}. \tag{S4-21}$$

The comparisons between theoretically-calculated and numerically-extracted trajectories are shown in Fig. S9 for several increasing w_0. As packet's width increases, the theoretical trajectory matches better with numerical one. The reason is that the spectrum width becomes narrower as the packet width

increases, making the approximation of Eq. (S4-17) less deviated from rigorous result in Eq. (S4-16). Since in experiment we have chosen $\beta = \pi/15$, for w_0 = 10, 15, 20, 30, we can obtain $|v_0| = |1/(\beta w_0)^2|$ = 0.228, 0.101, 0.057, 0.025 << 1. It is thus safe to omit v_0 in the calculation of d in Eq. (13) to get $d = -W/(1+v)$. The reason why we choose w_0 = 10 in experiment rather than larger width is due to the technique limitations for generating very broad Bloch-wave packet, see Methods for more details.

Section 5: Application examples with moving boundaries/potentials.

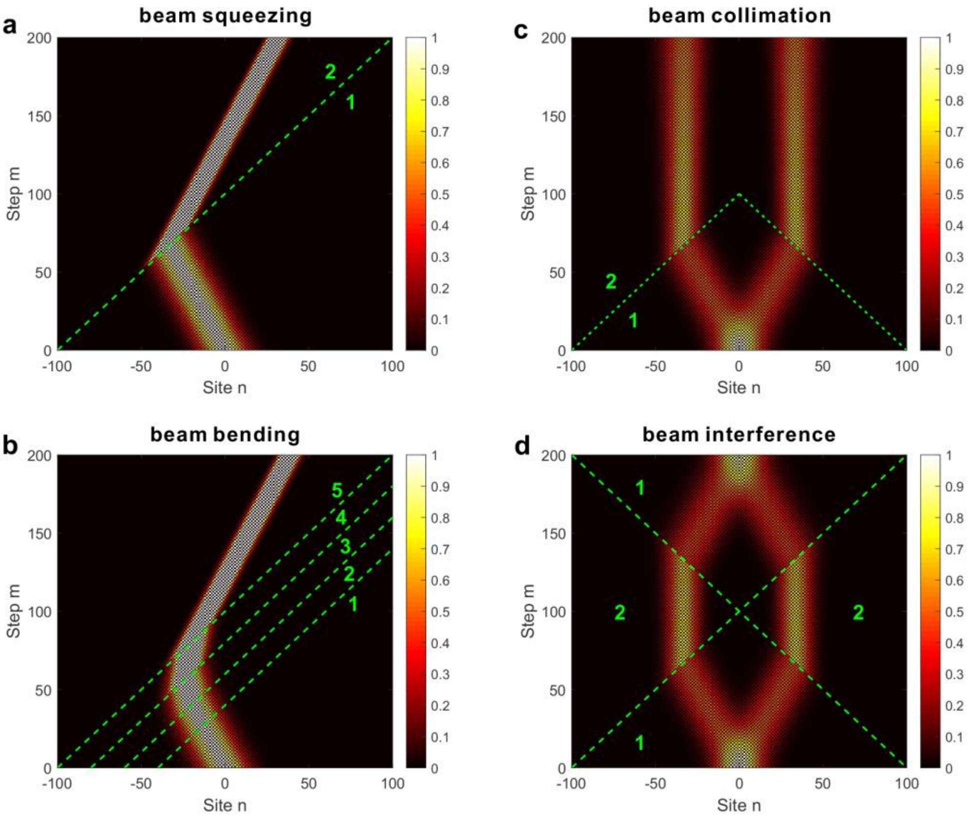

Fig. S10| Simulated examples of beam-control applications with moving boundaries/potentials. **a**, The gauge-potential distribution is $(\varphi, A_1) = (0, 0)$, $(\Delta\varphi, \Delta A) = (-\pi/2, -\pi/2)$ in region 1 and 2 with $\Delta\varphi + v\Delta A = -\pi$, which is the same with Fig. 3 in the main text. **b**, The gauge-potential distribution is $(\varphi_i, A_i) = [-(i-1)\pi/4, 0]$, i =1, 2, 3, 4 and 5, showing equal interval of scalar potential in adjacent regions. In **a** and **b**, the incident packet has a carrier Bloch momentum $k_{1,+} = -\pi/2$ and width $w_0 = 20$, which is excited from positive miniband. **c**, **d**, The gauge potential distribution is $(\varphi_1, A_1) = (0, 0)$, $(\Delta\varphi, \Delta A) = (-2\pi/3, 0)$ and $\Delta\varphi + v\Delta A = -2\pi/3$ to realize beam collimation. In **c** and **d**, we excite from both positive and negative minibands with $(U_0, V_0) = (1, 1)$ to get the beam splitting in the incident region.

In this supplementary section, we suggest several beam-control applications by using moving boundaries/potentials. Firstly, we apply the simplest single moving boundary to realize beam squeezing during one refraction process (Fig. S10a). By cascading multiple moving barriers with tailored gauge potential distributions and widths, we further achieve beam bending effect through multiple refraction processes (Fig. S10b). In Figs. S10c and S10d, we use combined moving potential boundaries to obtain the specific gauge potential distributions and realize both beam collimation and interference effects, respectively.
